# 3-(4-Methoxy­phen­yl)-6-(phenyl­sulfon­yl)perhydro-1,3-thiazolo[3′,4′:1,2]pyrrolo[4,5-*c*]pyrrole

**DOI:** 10.1107/S1600536808002134

**Published:** 2008-01-25

**Authors:** S. Sundaramoorthy, D. Gayathri, D. Velmurugan, K. Ravikumar, M. Poornachandran

**Affiliations:** a Centre of Advanced Study in Crystallography and Biophysics, University of Madras, Guindy Campus, Chennai 600025, India; b Laboratory of X-ray Crystallography, Indian Institute of Chemical Technology, Hyderabad 500007, India; c Department of Organic Chemistry, University of Madras, Guindy Campus, Chennai 600025, India

## Abstract

In the title compound, C_21_H_24_N_2_O_3_S_2_, the three five-membered rings adopt envelope conformations. The dihedral angle between the two aromatic rings is 68.4 (1)°. C—H⋯O inter­actions link the mol­ecules into a chain and the chains are cross-linked *via* C—H⋯π inter­actions involving the meth­oxy­phenyl ring.

## Related literature

For puckering parameters, see: Cremer & Pople (1975 ▶). For asymmetry parameters, see: Nardelli (1983 ▶). For general background, see: Amal Raj *et al.* (2003 ▶); Tsuru *et al.* (1988 ▶). For a related structure, see: Kavitha *et al.* (2006 ▶).
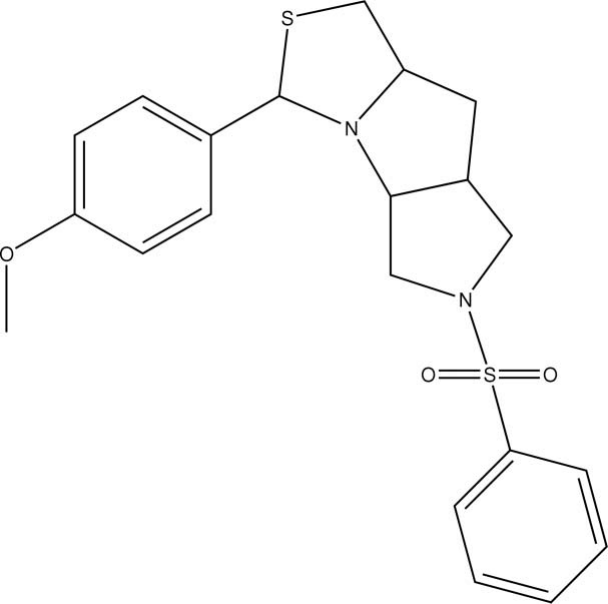


## Experimental

### 

#### Crystal data

C_21_H_24_N_2_O_3_S_2_
*M* *_r_* = 416.54
Monoclinic, 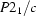

*a* = 14.5533 (8) Å
*b* = 8.3319 (5) Å
*c* = 16.8828 (9) Å
β = 98.923 (1)°
*V* = 2022.4 (2) Å^3^
*Z* = 4
Mo *K*α radiation
μ = 0.29 mm^−1^
*T* = 293 (2) K
0.24 × 0.23 × 0.21 mm

#### Data collection

Bruker SMART APEX CCD area-detector diffractometer
Absorption correction: none
22482 measured reflections
4769 independent reflections
3991 reflections with *I* > 2σ(*I*)
*R*_int_ = 0.020

#### Refinement

*R*[*F* ^2^ > 2σ(*F* ^2^)] = 0.048
*wR*(*F* ^2^) = 0.137
*S* = 1.00
4769 reflections
254 parameters
H-atom parameters constrained
Δρ_max_ = 0.46 e Å^−3^
Δρ_min_ = −0.16 e Å^−3^

### 

Data collection: *SMART* (Bruker, 2001 ▶); cell refinement: *SAINT* (Bruker, 2001 ▶); data reduction: *SAINT*; program(s) used to solve structure: *SHELXS97* (Sheldrick, 2008 ▶); program(s) used to refine structure: *SHELXL97* (Sheldrick, 2008 ▶); molecular graphics: *PLATON* (Spek, 2003 ▶); software used to prepare material for publication: *SHELXL97* and *PARST* (Nardelli, 1995 ▶).

## Supplementary Material

Crystal structure: contains datablocks I, global. DOI: 10.1107/S1600536808002134/ci2542sup1.cif

Structure factors: contains datablocks I. DOI: 10.1107/S1600536808002134/ci2542Isup2.hkl

Additional supplementary materials:  crystallographic information; 3D view; checkCIF report

## Figures and Tables

**Table 1:** Hydrogen-bond geometry (Å, °) *Cg*1 is the centroid of the C9–C14 ring.

*D*—H⋯*A*	*D*—H	H⋯*A*	*D*⋯*A*	*D*—H⋯*A*
C18—H18⋯O1^i^	0.93	2.56	3.437 (3)	158
C3—H3⋯*Cg*1^ii^	0.98	2.76	3.729 (2)	172

Symmetry codes: (i) 
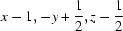
; (ii) 
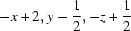
.

**Fig. 1.**
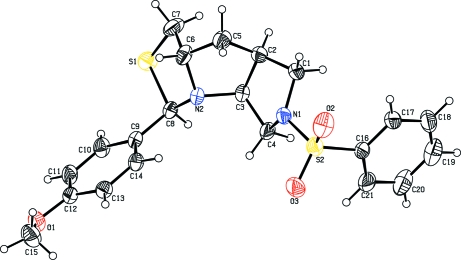
The molecular structure of the title compound, showing 30% probability displacement ellipsoids.

**Fig. 2.**
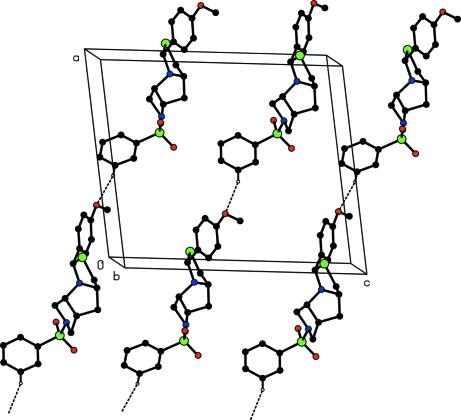
The crystal packing of the title compound viewed down the b axis.

## supplementary crystallographic information

### Comment

Substituted pyrrolidine compounds have gained much importance as they are the structural elements of many alkaloids. The pyrrolidine derivatives have been found to possess antifungal activity against various pathogens (Amal Raj *et al.*, 2003). Thiazolidine derivatives may act as potent inhibitors specific for Pro1yl Endopeptidase (Tsuru *et al.*, 1988). In view of the above facts, we have undertaken the X-ray crystal structure determination of the title compound.

Bond lenghts and angles in the title molecule (Fig. 1) are comparable to those observed in a related structure (Kavitha *et al.*, 2006). The sums of the bond angles around N1 (343.7°) and N2 (333.1°) indicate *sp*^3^-hybridization. The thiazolidine and the two pyrrolidine rings (N1/C1—C4, A, and C2/C3/N2/C5/C6, B) adopt envelope conformations. Atom N1 in ring A lies 0.597 (2) Å below the C1—C4 mean plane and atom C6 in ring B lies 0.563 (3) Å above the N2/C3/C2/C5 plane. In the thiazolidine ring, atom C6 deviates by 0.554 (3) Å from the plane of the rest of the atoms in the ring. The puckering parameters (Cremer & Pople, 1975) and the smallest displacement asymmetry parameters (Nardelli, 1983) are q_2_ = 0.406 (2) Å, φ = 187.3 (3)° and Δ_s_(N_1_) = 6.5 (2)° for ring A, q_2_ = 0.372 (2) Å, φ = 137.0 (3)° and Δ_s_(C_6_) = 4.2 (2)° for ring B, and q_2_ = 0.378 (2) Å, φ = 69.8 (3)° and Δ_s_(C_6_) = 3.7 (2)° for the thiazolidine ring. The dihedral angle between the two aromatic rings is 68.4 (1)°.

The crystal packing is stabilized by C—H···O and C—H···π intermolecular interactions.

### Experimental

A mixture of 2-(*N*-allyl-*N*-phenylsulfonyl) butanal (1.0 mmol) and of 2-*p*-methoxyphenylthiazolidine-4-carboxylic acid (1.5 mmol) in dry toluene (30 ml) was refluxed under Dean-Stark conditions till the completion of the reaction (3 h). The reaction mixture was then concentrated under reduced pressure. The residue was extracted with dichloromethane (2× 20 ml) and water (2× 20 ml). The organic layer was washed with brine solution (2× 20 ml), dried over anhydrous sodium sulfate and concentrated in vacuum. The residue was then subjected to column chromatography (silica gel, 100–200 mesh) with hexane-ethylacetate (8:2) to obtain the cycloadduct. Single crystals were obtained by recrystallization from methanol.

### Refinement

H atoms were included in calculated positions and treated in the subsequent refinement as riding atoms, with C—H = 0.93–0.98 Å and *U*_iso_(H) = 1.2–1.5(methyl) *U*_eq_(C).

### Crystal data

**Table:**

C_21_H_24_N_2_O_3_S_2_	*F*_000_ = 880
*M**_r_* = 416.54	*D*_x_ = 1.368 Mg m^−^^3^
Monoclinic, *P*2_1_/*c*	Mo *K*α radiation λ = 0.71073 Å
Hall symbol: -P 2ybc	Cell parameters from 2394 reflections
*a* = 14.5533 (8) Å	θ = 2.4–28.0º
*b* = 8.3319 (5) Å	µ = 0.29 mm^−^^1^
*c* = 16.8828 (9) Å	*T* = 293 (2) K
β = 98.923 (1)º	Block, pale yellow
*V* = 2022.4 (2) Å^3^	0.24 × 0.23 × 0.21 mm
*Z* = 4	

### Data collection

**Table:**

Bruker SMART APEX CCD area-detector diffractometer	3991 reflections with *I* > 2σ(*I*)
Radiation source: fine-focus sealed tube	*R*_int_ = 0.020
Monochromator: graphite	θ_max_ = 28.0º
*T* = 293(2) K	θ_min_ = 2.4º
ω scans	*h* = −18→18
Absorption correction: none	*k* = −10→11
22482 measured reflections	*l* = −21→22
4769 independent reflections	

### Refinement

**Table:**

Refinement on *F*^2^	Secondary atom site location: difference Fourier map
Least-squares matrix: full	Hydrogen site location: inferred from neighbouring sites
*R*[*F*^2^ > 2σ(*F*^2^)] = 0.048	H-atom parameters constrained
*wR*(*F*^2^) = 0.137	*w* = 1/[σ^2^(*F*_o_^2^) + (0.082*P*)^2^ + 0.473*P*] where *P* = (*F*_o_^2^ + 2*F*_c_^2^)/3
*S* = 1.00	(Δ/σ)_max_ = 0.001
4769 reflections	Δρ_max_ = 0.46 e Å^−^^3^
254 parameters	Δρ_min_ = −0.16 e Å^−^^3^
Primary atom site location: structure-invariant direct methods	Extinction correction: none

### Special details

**Table:**

Geometry. All e.s.d.'s (except the e.s.d. in the dihedral angle between two l.s. planes) are estimated using the full covariance matrix. The cell e.s.d.'s are taken into account individually in the estimation of e.s.d.'s in distances, angles and torsion angles; correlations between e.s.d.'s in cell parameters are only used when they are defined by crystal symmetry. An approximate (isotropic) treatment of cell e.s.d.'s is used for estimating e.s.d.'s involving l.s. planes.
Refinement. Refinement of *F*^2^ against ALL reflections. The weighted *R*-factor *wR* and goodness of fit *S* are based on *F*^2^, conventional *R*-factors *R* are based on *F*, with *F* set to zero for negative *F*^2^. The threshold expression of *F*^2^ > σ(*F*^2^) is used only for calculating *R*-factors(gt) *etc*. and is not relevant to the choice of reflections for refinement. *R*-factors based on *F*^2^ are statistically about twice as large as those based on *F*, and *R*- factors based on ALL data will be even larger.

### Fractional atomic coordinates and isotropic or equivalent isotropic displacement parameters (Å^2^)

**Table:**

	*x*	*y*	*z*	*U*_iso_*/*U*_eq_	
C1	0.65354 (11)	0.0421 (2)	0.27798 (12)	0.0543 (4)	
H1A	0.6206	0.0086	0.2262	0.065*	
H1B	0.6116	0.0371	0.3173	0.065*	
C2	0.74011 (12)	−0.0593 (2)	0.30329 (11)	0.0518 (4)	
H2	0.7317	−0.1681	0.2813	0.062*	
C3	0.81895 (10)	0.0293 (2)	0.26945 (9)	0.0439 (3)	
H3	0.8425	−0.0356	0.2285	0.053*	
C4	0.77552 (11)	0.1850 (2)	0.23356 (11)	0.0526 (4)	
H4A	0.8180	0.2747	0.2453	0.063*	
H4B	0.7576	0.1760	0.1760	0.063*	
C5	0.77266 (13)	−0.0635 (3)	0.39390 (11)	0.0626 (5)	
H5A	0.7481	0.0270	0.4201	0.075*	
H5B	0.7534	−0.1622	0.4171	0.075*	
C6	0.87749 (12)	−0.0543 (3)	0.40161 (10)	0.0569 (4)	
H6	0.9046	−0.0132	0.4545	0.068*	
C7	0.92284 (15)	−0.2141 (3)	0.38422 (15)	0.0727 (6)	
H7A	0.8825	−0.2742	0.3436	0.087*	
H7B	0.9354	−0.2788	0.4325	0.087*	
C8	0.98632 (10)	0.04673 (19)	0.32061 (9)	0.0436 (3)	
H8	0.9840	0.0590	0.2626	0.052*	
C9	1.05361 (11)	0.16890 (19)	0.36222 (9)	0.0436 (3)	
C10	1.14935 (12)	0.1485 (2)	0.36544 (12)	0.0550 (4)	
H10	1.1713	0.0577	0.3423	0.066*	
C11	1.21182 (12)	0.2592 (2)	0.40186 (11)	0.0564 (4)	
H11	1.2752	0.2426	0.4032	0.068*	
C12	1.18109 (11)	0.3950 (2)	0.43660 (9)	0.0479 (4)	
C13	1.08652 (11)	0.4217 (2)	0.43145 (10)	0.0494 (4)	
H13	1.0649	0.5149	0.4526	0.059*	
C14	1.02415 (11)	0.3086 (2)	0.39456 (10)	0.0478 (4)	
H14	0.9607	0.3274	0.3915	0.057*	
C15	1.22176 (17)	0.6174 (3)	0.52376 (16)	0.0805 (6)	
H15A	1.1852	0.6967	0.4918	0.121*	
H15B	1.2763	0.6669	0.5529	0.121*	
H15C	1.1857	0.5707	0.5608	0.121*	
C16	0.56879 (11)	0.3179 (2)	0.15034 (11)	0.0500 (4)	
C17	0.48978 (12)	0.2243 (2)	0.13751 (13)	0.0618 (5)	
H17	0.4622	0.1885	0.1804	0.074*	
C18	0.45196 (16)	0.1844 (3)	0.05922 (17)	0.0806 (7)	
H18	0.3991	0.1203	0.0497	0.097*	
C19	0.4920 (2)	0.2388 (4)	−0.00370 (16)	0.0903 (8)	
H19	0.4664	0.2110	−0.0558	0.108*	
C20	0.56928 (18)	0.3335 (4)	0.00945 (16)	0.0913 (8)	
H20	0.5956	0.3713	−0.0337	0.110*	
C21	0.60859 (14)	0.3736 (3)	0.08689 (14)	0.0717 (6)	
H21	0.6615	0.4377	0.0959	0.086*	
N1	0.69349 (9)	0.20342 (17)	0.27399 (8)	0.0463 (3)	
N2	0.89254 (8)	0.05860 (16)	0.33850 (7)	0.0425 (3)	
O1	1.24842 (9)	0.49627 (18)	0.47326 (9)	0.0655 (4)	
O2	0.55465 (11)	0.3473 (2)	0.30071 (10)	0.0803 (5)	
O3	0.68031 (11)	0.49379 (17)	0.24897 (11)	0.0845 (5)	
S1	1.02851 (3)	−0.16174 (6)	0.34950 (3)	0.06126 (16)	
S2	0.62353 (3)	0.35418 (5)	0.24924 (3)	0.05671 (16)	

### Atomic displacement parameters (Å^2^)

**Table:**

	*U*^11^	*U*^22^	*U*^33^	*U*^12^	*U*^13^	*U*^23^
C1	0.0391 (8)	0.0524 (9)	0.0690 (11)	−0.0053 (7)	0.0012 (7)	0.0057 (8)
C2	0.0474 (9)	0.0442 (8)	0.0619 (10)	−0.0017 (7)	0.0025 (7)	0.0016 (7)
C3	0.0408 (7)	0.0513 (9)	0.0379 (7)	0.0052 (6)	0.0013 (6)	−0.0012 (6)
C4	0.0375 (8)	0.0647 (11)	0.0543 (9)	0.0039 (7)	0.0033 (7)	0.0166 (8)
C5	0.0539 (10)	0.0767 (13)	0.0591 (10)	0.0051 (9)	0.0148 (8)	0.0172 (9)
C6	0.0501 (9)	0.0787 (13)	0.0418 (8)	0.0110 (9)	0.0065 (7)	0.0119 (8)
C7	0.0654 (12)	0.0688 (13)	0.0853 (14)	0.0174 (10)	0.0158 (10)	0.0367 (11)
C8	0.0414 (7)	0.0472 (8)	0.0412 (7)	0.0091 (6)	0.0033 (6)	0.0006 (6)
C9	0.0405 (8)	0.0486 (8)	0.0402 (7)	0.0083 (6)	0.0018 (6)	0.0033 (6)
C10	0.0434 (8)	0.0551 (10)	0.0650 (10)	0.0143 (7)	0.0035 (7)	−0.0071 (8)
C11	0.0369 (8)	0.0617 (11)	0.0681 (11)	0.0100 (7)	0.0006 (7)	−0.0002 (9)
C12	0.0454 (8)	0.0520 (9)	0.0441 (8)	0.0009 (7)	0.0005 (6)	0.0052 (7)
C13	0.0495 (9)	0.0479 (9)	0.0507 (9)	0.0068 (7)	0.0080 (7)	−0.0013 (7)
C14	0.0380 (7)	0.0537 (9)	0.0513 (9)	0.0088 (7)	0.0052 (6)	−0.0007 (7)
C15	0.0727 (13)	0.0751 (14)	0.0901 (16)	−0.0104 (12)	0.0017 (12)	−0.0244 (12)
C16	0.0384 (8)	0.0460 (8)	0.0629 (10)	0.0071 (6)	−0.0007 (7)	0.0056 (7)
C17	0.0448 (9)	0.0581 (11)	0.0795 (13)	−0.0016 (8)	−0.0002 (8)	0.0085 (9)
C18	0.0584 (12)	0.0700 (13)	0.1027 (18)	−0.0003 (10)	−0.0211 (12)	−0.0081 (13)
C19	0.0831 (17)	0.108 (2)	0.0723 (14)	0.0324 (15)	−0.0127 (12)	−0.0067 (14)
C20	0.0679 (14)	0.135 (2)	0.0702 (14)	0.0243 (15)	0.0080 (11)	0.0286 (14)
C21	0.0458 (9)	0.0839 (14)	0.0830 (14)	0.0029 (9)	0.0022 (9)	0.0269 (12)
N1	0.0369 (6)	0.0462 (7)	0.0539 (7)	0.0004 (5)	0.0009 (5)	−0.0001 (6)
N2	0.0384 (6)	0.0499 (7)	0.0376 (6)	0.0061 (5)	0.0012 (5)	−0.0003 (5)
O1	0.0506 (7)	0.0672 (8)	0.0754 (9)	−0.0045 (6)	−0.0007 (6)	−0.0105 (7)
O2	0.0724 (9)	0.0955 (12)	0.0742 (9)	0.0299 (8)	0.0151 (7)	−0.0118 (8)
O3	0.0785 (10)	0.0448 (7)	0.1191 (13)	−0.0049 (7)	−0.0199 (9)	−0.0081 (8)
S1	0.0558 (3)	0.0487 (3)	0.0802 (3)	0.01351 (19)	0.0134 (2)	−0.0004 (2)
S2	0.0506 (3)	0.0477 (3)	0.0683 (3)	0.00750 (18)	−0.0018 (2)	−0.00786 (19)

### Geometric parameters (Å, °)

**Table:**

C1—N1	1.470 (2)	C10—C11	1.372 (3)
C1—C2	1.522 (2)	C10—H10	0.93
C1—H1A	0.97	C11—C12	1.381 (3)
C1—H1B	0.97	C11—H11	0.93
C2—C5	1.530 (3)	C12—O1	1.367 (2)
C2—C3	1.546 (2)	C12—C13	1.384 (2)
C2—H2	0.98	C13—C14	1.387 (2)
C3—N2	1.4760 (18)	C13—H13	0.93
C3—C4	1.527 (2)	C14—H14	0.93
C3—H3	0.98	C15—O1	1.413 (3)
C4—N1	1.472 (2)	C15—H15A	0.96
C4—H4A	0.97	C15—H15B	0.96
C4—H4B	0.97	C15—H15C	0.96
C5—C6	1.513 (2)	C16—C21	1.375 (3)
C5—H5A	0.97	C16—C17	1.378 (2)
C5—H5B	0.97	C16—S2	1.7611 (18)
C6—N2	1.463 (2)	C17—C18	1.391 (3)
C6—C7	1.534 (3)	C17—H17	0.93
C6—H6	0.98	C18—C19	1.366 (4)
C7—S1	1.783 (2)	C18—H18	0.93
C7—H7A	0.97	C19—C20	1.364 (4)
C7—H7B	0.97	C19—H19	0.93
C8—N2	1.4461 (19)	C20—C21	1.384 (4)
C8—C9	1.508 (2)	C20—H20	0.93
C8—S1	1.8812 (16)	C21—H21	0.93
C8—H8	0.98	N1—S2	1.630 (1)
C9—C14	1.382 (2)	O2—S2	1.426 (2)
C9—C10	1.396 (2)	O3—S2	1.427 (2)
			
N1—C1—C2	101.78 (13)	C11—C10—H10	119.2
N1—C1—H1A	111.4	C9—C10—H10	119.2
C2—C1—H1A	111.4	C10—C11—C12	120.36 (15)
N1—C1—H1B	111.4	C10—C11—H11	119.8
C2—C1—H1B	111.4	C12—C11—H11	119.8
H1A—C1—H1B	109.3	O1—C12—C11	116.22 (15)
C1—C2—C5	114.08 (16)	O1—C12—C13	124.43 (16)
C1—C2—C3	105.12 (13)	C11—C12—C13	119.34 (16)
C5—C2—C3	104.36 (13)	C12—C13—C14	119.61 (16)
C1—C2—H2	111.0	C12—C13—H13	120.2
C5—C2—H2	111.0	C14—C13—H13	120.2
C3—C2—H2	111.0	C9—C14—C13	121.87 (15)
N2—C3—C4	112.20 (14)	C9—C14—H14	119.1
N2—C3—C2	106.04 (12)	C13—C14—H14	119.1
C4—C3—C2	105.49 (12)	O1—C15—H15A	109.5
N2—C3—H3	111.0	O1—C15—H15B	109.5
C4—C3—H3	111.0	H15A—C15—H15B	109.5
C2—C3—H3	111.0	O1—C15—H15C	109.5
N1—C4—C3	102.71 (13)	H15A—C15—H15C	109.5
N1—C4—H4A	111.2	H15B—C15—H15C	109.5
C3—C4—H4A	111.2	C21—C16—C17	120.69 (19)
N1—C4—H4B	111.2	C21—C16—S2	119.82 (15)
C3—C4—H4B	111.2	C17—C16—S2	119.33 (15)
H4A—C4—H4B	109.1	C16—C17—C18	118.8 (2)
C6—C5—C2	103.68 (14)	C16—C17—H17	120.6
C6—C5—H5A	111.0	C18—C17—H17	120.6
C2—C5—H5A	111.0	C19—C18—C17	120.4 (2)
C6—C5—H5B	111.0	C19—C18—H18	119.8
C2—C5—H5B	111.0	C17—C18—H18	119.8
H5A—C5—H5B	109.0	C18—C19—C20	120.4 (2)
N2—C6—C5	103.46 (13)	C18—C19—H19	119.8
N2—C6—C7	107.54 (14)	C20—C19—H19	119.8
C5—C6—C7	113.43 (19)	C19—C20—C21	120.2 (2)
N2—C6—H6	110.7	C19—C20—H20	119.9
C5—C6—H6	110.7	C21—C20—H20	119.9
C7—C6—H6	110.7	C16—C21—C20	119.5 (2)
C6—C7—S1	105.61 (15)	C16—C21—H21	120.3
C6—C7—H7A	110.6	C20—C21—H21	120.3
S1—C7—H7A	110.6	C1—N1—C4	106.32 (14)
C6—C7—H7B	110.6	C1—N1—S2	118.72 (10)
S1—C7—H7B	110.6	C4—N1—S2	118.72 (11)
H7A—C7—H7B	108.8	C8—N2—C6	111.15 (12)
N2—C8—C9	114.97 (13)	C8—N2—C3	114.61 (12)
N2—C8—S1	106.84 (11)	C6—N2—C3	107.25 (13)
C9—C8—S1	109.88 (10)	C12—O1—C15	117.99 (15)
N2—C8—H8	108.3	C7—S1—C8	92.74 (8)
C9—C8—H8	108.3	O3—S2—O2	119.8 (1)
S1—C8—H8	108.3	O3—S2—N1	106.9 (1)
C14—C9—C10	117.13 (16)	O2—S2—N1	106.4 (1)
C14—C9—C8	122.25 (14)	O3—S2—C16	108.3 (1)
C10—C9—C8	120.52 (14)	O2—S2—C16	108.3 (1)
C11—C10—C9	121.58 (16)	N1—S2—C16	106.5 (1)
			
N1—C1—C2—C5	84.89 (18)	C19—C20—C21—C16	−0.4 (4)
N1—C1—C2—C3	−28.83 (17)	C2—C1—N1—C4	43.59 (17)
C1—C2—C3—N2	124.51 (14)	C2—C1—N1—S2	−179.41 (12)
C5—C2—C3—N2	4.15 (18)	C3—C4—N1—C1	−40.24 (16)
C1—C2—C3—C4	5.32 (18)	C3—C4—N1—S2	−177.24 (11)
C5—C2—C3—C4	−115.04 (15)	C9—C8—N2—C6	−96.95 (16)
N2—C3—C4—N1	−94.75 (15)	S1—C8—N2—C6	25.25 (15)
C2—C3—C4—N1	20.26 (17)	C9—C8—N2—C3	141.25 (14)
C1—C2—C5—C6	−140.00 (16)	S1—C8—N2—C3	−96.54 (13)
C3—C2—C5—C6	−25.83 (19)	C5—C6—N2—C8	−162.77 (15)
C2—C5—C6—N2	38.5 (2)	C7—C6—N2—C8	−42.48 (19)
C2—C5—C6—C7	−77.72 (19)	C5—C6—N2—C3	−36.79 (18)
N2—C6—C7—S1	39.16 (19)	C7—C6—N2—C3	83.51 (17)
C5—C6—C7—S1	152.93 (13)	C4—C3—N2—C8	−101.22 (15)
N2—C8—C9—C14	−18.7 (2)	C2—C3—N2—C8	144.10 (13)
S1—C8—C9—C14	−139.26 (14)	C4—C3—N2—C6	134.88 (14)
N2—C8—C9—C10	164.94 (15)	C2—C3—N2—C6	20.21 (17)
S1—C8—C9—C10	44.39 (19)	C11—C12—O1—C15	−166.82 (19)
C14—C9—C10—C11	2.5 (3)	C13—C12—O1—C15	14.2 (3)
C8—C9—C10—C11	178.99 (17)	C6—C7—S1—C8	−21.16 (15)
C9—C10—C11—C12	0.0 (3)	N2—C8—S1—C7	−1.24 (13)
C10—C11—C12—O1	178.36 (17)	C9—C8—S1—C7	124.11 (13)
C10—C11—C12—C13	−2.6 (3)	C1—N1—S2—O3	−179.18 (14)
O1—C12—C13—C14	−178.35 (16)	C4—N1—S2—O3	−47.46 (16)
C11—C12—C13—C14	2.7 (3)	C1—N1—S2—O2	51.72 (16)
C10—C9—C14—C13	−2.4 (3)	C4—N1—S2—O2	−176.55 (13)
C8—C9—C14—C13	−178.82 (15)	C1—N1—S2—C16	−63.61 (15)
C12—C13—C14—C9	−0.2 (3)	C4—N1—S2—C16	68.11 (14)
C21—C16—C17—C18	1.3 (3)	C21—C16—S2—O3	24.79 (18)
S2—C16—C17—C18	−174.25 (15)	C17—C16—S2—O3	−159.64 (15)
C16—C17—C18—C19	−0.7 (3)	C21—C16—S2—O2	156.12 (16)
C17—C18—C19—C20	−0.3 (4)	C17—C16—S2—O2	−28.32 (18)
C18—C19—C20—C21	0.9 (4)	C21—C16—S2—N1	−89.80 (17)
C17—C16—C21—C20	−0.7 (3)	C17—C16—S2—N1	85.77 (16)
S2—C16—C21—C20	174.78 (18)		

### Hydrogen-bond geometry (Å, °)

**Table:**

*D*—H···*A*	*D*—H	H···*A*	*D*···*A*	*D*—H···*A*
C18—H18···O1^i^	0.93	2.56	3.437 (3)	158
C3—H3···Cg1^ii^	0.98	2.76	3.729 (2)	172

Symmetry codes: (i) *x*−1, −*y*+1/2, *z*−1/2; (ii) −*x*+2, *y*−1/2, −*z*+1/2.
